# Exploring medication safety structures and processes in nursing homes: a cross-sectional study

**DOI:** 10.1007/s11096-023-01625-6

**Published:** 2023-08-10

**Authors:** Lauriane Favez, Franziska Zúñiga, Carla Meyer-Massetti

**Affiliations:** 1 Pflegewissenschaft – Nursing Science, Department Public Health, Faculty of Medicine, University of Basel, Basel, Switzerland; 2 School of Engineering and Management Vaud, HES-SO University of Applied Sciences and Arts, Western Switzerland, Yverdon-les-Bains, Switzerland; 3 Clinical Pharmacy and Epidemiology, Department of Pharmaceutical Sciences, University of Basel, Basel, Switzerland; 4 Institute for Primary Healthcare BIHAM, University of Bern, Bern, Switzerland; 5 Clinical Pharmacology and Toxicology, Department of General Internal Medicine, Clinic for General Internal Medicine, Inselspital - University Hospital of Bern, Bern, Switzerland

**Keywords:** Clinical pharmacy, Interprofessional collaboration, Medication safety, Nursing homes

## Abstract

**Background:**

Medication safety is important to limit adverse events for nursing home residents. Several factors, such as interprofessional collaboration with pharmacists and medication reviews, have been shown in the literature to influence medication safety processes.

**Aim:**

This study had three main objectives: (1) To assess how facility- and unit-level organization and infrastructure are related to medication use processes; (2) To determine the extent of medication safety-relevant processes; and (3) To explore pharmacies’ and pharmacists’ involvement in nursing homes’ medication-related processes.

**Method:**

Cross-sectional multicenter survey data (2018–2019) from a convenience sample of 118 Swiss nursing homes were used. Data were collected on facility and unit characteristics, pharmacy services, as well as medication safety-related structures and processes. Descriptive statistics were used.

**Results:**

Most of the participating nursing homes (93.2%) had electronic resident health record systems that supported medication safety in various ways (e.g., medication lists, interaction checks). Electronic data exchanges with outside partners such as pharmacies or physicians were available for fewer than half (10.2–46.3%, depending on the partner). Pharmacists collaborating with nursing homes were mainly involved in logistical support. Medication reviews were reportedly conducted regularly in two-thirds of facilities.

**Conclusion:**

A high proportion of Swiss nursing homes have implemented diverse processes and structures that support medication use and safety for residents; however, their collaboration with pharmacists remains relatively limited.

## Impact statements

- Electronic data exchanges with external partners (e.g., pharmacies, primary care physicians) differ widely between language regions in Switzerland.
- Increasing opportunities to jointly access data, or at least increasing electronic communication, might benefit interprofessional collaboration and optimize both medication use practices and medication safety.
- More nursing homes were working with pharmacists than legally required, which suggests that interprofessional collaboration is increasingly deemed beneficial by nursing homes.
- Access to clinical support by pharmacists supports medication safety-related practices.
- Nurses might be valuable partners for pharmacists, facilitating interprofessional collaboration through the initiation of medication reviews.

## Introduction

As in many other countries, Switzerland’s proportion of elderly people is increasing rapidly. Between 2012 and 2017, the number of people between 65 and 79 years of age has grown by 24.5%. Of those aged 80 or older, the proportion has grown by 22% [1]. An estimated 1.5% of the Swiss population between the ages of 65 and 79 live in a nursing home (NH). For people over 80 years of age, that figure rises to 15.7% [2]. In comparison, in 2021, in OECD countries between 0.4% (Latvia) and 10.3% (Lithuania) of the population over 65 lived in institutions, with 5.2% in the USA and 3.9% in Switzerland [3]. While NH residents’ care needs depend more on their length of stay in the facility than on their age, multimorbidity and polypharmacy increase with age, magnifying their importance in this population [4–6].

Polypharmacy is most commonly defined as the regular application of five or more medications per day [7]. In the scope of the SHELTER study, assessing data of more than 4000 NH residents in eight European countries, polypharmacy of five or more medications was present in 49.7% of residents [8]. Individual studies reported even higher numbers. A cross-sectional study from Spain identified up to 78.8% of NH residents being on at least seven medications [9]. In Germany, up to 72 of patients needing care in an NH are polymedicated, compared to 56% living in their own home [10]. In 2017, a health insurance data-based report estimated that 85% of Swiss NH residents were polymedicated, receiving an average of 9.3 prescribed regular medications per day [11]. Two Swiss studies reported that respectively 43.0% [12] and 44.3% (range per facility: 21.9–69.0%) [13] of NH residents were taking nine or more active ingredients weekly. Polypharmacy is often associated with medication-related problems such as adverse drug reactions and medication errors (MEs) [14–16]. Depending on the type of staff asked (i.e., NH directors, ward leaders, staff members), satisfaction levels with polypharmacy management in Swiss NHs range between 49.2 and 57.2% [13]. By helping care staff avoid MEs and anticipate adverse drug reactions, a well-structured medication use process is important to ensure medication safety, an important aspect of resident safety [17].

Medication safety is defined as the combination of measures to ensure the appropriate and safe use of medications and addresses every aspect of the medication use process [18]. Therefore, optimization of medication-relevant activities should lead to decreases in adverse drug events, i.e., increases in resident safety [18]. In many Swiss cantons (i.e., regions), the authorization for facilities to store medications centrally requires a quality management system to regulate medication use processes. Such systems are often managed by pharmacists. In NHs, medication use processes are often complex, consisting of different part processes, involving multiple professions, as well as residents and even informal caregivers [19]. It encompasses not only prescribing—a key factor of appropriate polypharmacy—but also logistical concerns (i.e., ordering, delivery, storage, redistribution of medications) [20]. It also includes dispensing, preparing, administration, monitoring, documenting and communicating medication-related information during transition-of-care situations [20]. Gurwitz et al. showed that errors resulting in preventable adverse drug events occurred most often in the stages of ordering and monitoring; while errors in transcription, dispensing, and administration were less common [21].

Searching for the systemic roots of medication-related problems, Al-Jumaili et al. (2017) identified five categories of work system factors that affect NHs’ medication safety: persons (residents, staff), methods of organization, tools and technology, tasks, and the work environment [22]. Adequate staffing reduced preventable adverse events and organizational factors such as well-structured interprofessional collaboration as well as physician and pharmacist accessibility played essential roles in preventing medication errors [22]. While Swiss NHs have numerous approaches to collaboration with physicians, all fit into two broad categories [23]: (1) those that employ at least one in-house physician (who may also be responsible for the NH’s organizational tasks, e.g., medication supply), but also work with residents’ primary care physicians (free choice of physician is mandatory in Switzerland); or (2) those that do not employ in-house physicians, relying entirely on residents’ primary care physicians. Having on-site physicians impacts NHs’ collaboration with pharmacies and other primary care physicians (e.g., regarding medications administration) [23]. Not all Swiss NHs have a legal obligation to work with pharmacists; however, when they do, the pharmacist is legally responsible for all of their medication use and supply processes (except prescription and administration of medication, which are physicians’ responsibilities) as well as to maintain the medication supply [19].

Pharmacists can contribute substantially to residents’ care by implementing interventions that promote high-quality medication-related practices [24]. These include clinical pharmacy activities such as regular medication reviews, which flag potentially inappropriate medications (PIMs), thereby reducing inappropriate polypharmacy and adverse drug reactions [25–27]. Furthermore, timely and accurate medication use information reduces medication preparation and administration errors [28]. Pharmacists can also help optimize medication-relevant processes overall, e.g., by implementing and promoting technology such as electronic systems to support the medication use process [22].

### Aim

This study’s overall aim was to investigate medication safety structures and processes in nursing homes in Switzerland.

Based on current knowledge about factors influencing medication safety in NHs, this study had three main objectives: (1) to assess the organization and infrastructure at NH facility and unit level (= ward level) in connection with the medication use processes; (2) to determine the extent to which medication safety-relevant processes are in use in NHs; and (3) to explore pharmacies’ and pharmacists’ involvement in NHs’ medication-related processes.

### Ethics approval

This study was granted an ethics waiver from the responsible Swiss ethics committee, as it was an observational study and individuals’ data were collected anonymously (Northwest and Central Switzerland ethics committee, BASEC Nr Req-2018–00420 on June 5, 2018).

## Method

### Study design

This was a multicenter cross-sectional study using data from the Swiss Nursing Home Human Resources Project 2018 (SHURP 2018) [13]. SHURP 2018 was a research project aiming at investigating organizational and work environment factors and their association with quality of care in nursing homes.

### Setting and sample

The study included a convenience sample of NHs from two of Switzerland’s major language regions: the German- and the French-speaking parts. The sample consisted of NHs that had participated in the first SHURP project (2013–2015) [29] and who agreed to participate in the present study, along with randomly-selected NHs. In addition, we recruited from NH associations collaborating with our research team, and some NHs asked proactively to be included. Recruitment took place between December 2017 and March 2019. To be eligible for inclusion, each NH had to be recognized as such by the relevant regional authorities. The study sample also included care staff members and unit leaders. Staff members were eligible if they were working in direct resident care, understood either German or French, and had been working a minimum of 20% (one day/week) for a minimum of one month in their current nursing home unit. For this analysis, we included only registered nurses and licensed practical nurses. Unit leaders had to hold this position in their NH and understand German or French to be included.

### Variables and measurement

Facility (nursing home) and unit (ward) characteristics were collected using questionnaires for facility-level managers and unit leaders respectively. Facility and unit questionnaires used in the first edition of the study [29] were adapted based on experience and study’s needs. We collected information on facility characteristics (e.g., NH size, ownership, type of physician collaboration), any in-house pharmacy services (e.g., the specific role(s) of the pharmacist or responsible physician), as well as internal structures and processes regarding medication (e.g., medication use guidelines, the handling of medication reviews, the availability of clinical decision support systems, e.g., automated interaction checking tools). A medication review was defined as a comprehensive and structured analysis of individual residents’ current medications. Questionnaires were also distributed to care staff members and unit leaders. Care staff were asked about whether on their unit the number of medications a resident receives are checked, whether perceptions about too many or unnecessary medication use is discussed with physicians or pharmacists and whether staff suggestions regarding medication use are taken up by physicians. Items were 5-point Likert-type self-developed questions, with responses ranging from “strongly disagree” to “strongly agree”. Unit leaders were asked about medication processes using a self-developed question (5-point Likert-type answer: “strongly disagree” to “strongly agree”), as well as self-developed questions about triggers for medication reviews. All questions were developed based on review of the relevant literature and discussions within the research group. Questionnaires were distributed either in French or in German.

### Data collection

Between September 2018 and October 2019, participating NHs received questionnaires to collect facility and unit data, as well as care staff members and unit leaders’ data. To ensure respondents’ anonymity, each questionnaire was provided with a pre-stamped, pre-addressed envelope. Each NH director had previously given written consent for the NH’s participation. In addition to being assured that participation was entirely voluntary, all staff were provided full information about the nature of the study along with their questionnaires; therefore, returning the questionnaire was considered informed consent. Confidentiality was guaranteed and staff were informed they could withdraw consent at any point.

### Data analysis

Descriptive statistics including percentages or medians and interquartile ranges (IQRs) were calculated. Data analysis was performed using R version 4.0.2. [30]. As very few values were missing (< 5%), they were deleted pairwise. One variable (role of pharmacist) was dichotomized and the Chi-Square Test of Independence was used to determine its association with the others. The significance level was set at 0.05.

## Results

### Sample description

A total of 118 NHs participated in the study. Of these 118 NHs, 83.1% (*n* = 98) were situated in the German-speaking part of Switzerland, 72.0% (*n* = 85) were located in urban areas, and 45.8% (*n* = 54) were publicly owned. The average number of beds was 84. Further descriptive statistics are provided in Table 1. Furthermore, we included all 371 NH units (= wards) and 385 NH unit leaders in this analysis, as well as a sub-sample of 2413 registered nurses and licensed practical nurses out of 4442 care workers who participated in the study.

**Table 1:** Description of the participating nursing homes (*n* = 118)

Nursing homes characteristics	*n*	%
*Language region*		
German part	98	83.1
French part	20	16.9
*Ownership*		
Private	64	54.2
Public	54	45.8
Size (mean bed number (SD))	84	47.5
*Catchment area*		
Rural	10	8.5
Suburban	23	19.5
Urban	85	72.0
Physician system: at least one in-house physician	60	50.9

Catchment areas are defined according to the Swiss Federal Office of Statistics (www.bfs.admin.ch). Missing were 0, except for size which had *n* = 3 (2.5%) of missing data

### Objective 1: Medication use organization and infrastructure

Regarding the overall use of technology, 93.2% (*n* = 110) of participating facilities reported using an electronic resident record system. Publicly funded facilities reported a lower percentage of implementation (87.0%, *n* = 47) compared to privately-owned facilities (98.4%, *n* = 63). Electronic clinical decision support was available in the format of interaction checks in 70.3% of facilities (*n* = 83). Still, only a minority (43.1%, *n* = 47) of NH leaders stated that their electronic systems supported adequate prescription of medications. Electronic data exchange across care interfaces was most possible with NHs’ in-house physicians (46.3%, *n* = 50), followed by pharmacies (38.0%, *n* = 41) and external primary care practices (29.9%, *n* = 32). However, it was comparatively rare with labs (13.0%, *n* = 14) and hospitals (10.2%, *n* = 11). Disparities regarding the availability of electronic data exchange were reported across language regions, with NHs in the French-speaking part typically more advanced than those in the German speaking part regarding all partners. This was especially true for primary care physicians (22.7%, *n* = 20 in the German-speaking part versus 63.2%, *n* = 12 in the French-speaking part) and pharmacies (31.5%, *n* = 28 for the German-speaking part, 68.4%, *n* = 13 for the French-speaking part).

### Objective 2: Medication-safety characteristics and processes

The majority of NHs (91.5%, *n* = 108) had guidelines in place regulating and structuring their medication use processes. However, only 48.3% of unit leaders (*n* = 186) reported that processes were in place to systematically verify polypharmacy and regularly adjust the number of medications administered to residents. Some facilities had additional instruments that might contribute to medication safety, including standardized medication lists for priority prescribing (23.7%, *n* = 28) and for PIM identification (12.7%, *n* = 15).

In 88.1% (*n* = 104) of participating facilities, a guideline for the execution of medication reviews was available; however, according to NH leaders, only 66.1% (*n* = 78) of facilities regularly conducted medication reviews, with a median of two medication reviews per resident per year. According to unit leaders, medication reviews mainly took place during rounds (91.4%, *n* = 352), but were also initiated following an incident (76.1%, *n* = 293), as a part of routine assessments (41.3%, *n* = 159) and within the scope of specialized geriatric assessments (12.7%, *n* = 49). According to NH leaders, they were predominantly initiated by a registered nurse (53.9%, *n* = 41), followed by a pharmacist (36.4%, *n* = 28), then a physician (29.9%, *n* = 23) and a nurse expert (28.0%, *n* = 21).

### Objective 3: Pharmacy and pharmacist involvement in medication-related processes

Most NHs (72.9%, *n* = 86) worked with local external pharmacies for drug provision to their facilities and/or residents and worked with a median of one pharmacy. A majority (87.1%, *n* = 101) also worked with consultant pharmacists, although only 69.2% (*n* = 81) were legally obliged to do so. Further, 84.7% (*n* = 100) reported pharmacists’ involvement in logistical tasks (e.g., ensuring adequate supplies and storage of medication); and 27.0% (*n* = 27) of these NHs reported that their pharmacists had no other roles within the NH. Pharmacists fulfilled clinical roles in 51.7% (*n* = 61) of NHs and educational roles in 43.2% (*n* = 51).

Regarding interprofessional collaboration with pharmacists and inappropriate polypharmacy, 77.0% (*n* = 1837) of care staff members reported discussions with their pharmacists or physicians about whether particular residents were receiving too many medications, 59.7% (*n* = 1421) believed physicians adequately reflected on care team suggestions, and 57.1% (*n* = 1362) perceived that the staff was paying attention to the number of medications given to residents.

Finally, we compared NH medication use processes based on pharmacists’ roles: NHs working with pharmacists in clinical roles had more processes and structures in place regarding medication use and safety than those who did not. For instance, while 21.3% of NHs (*n* = 13) with pharmacists performing clinical roles had lists of inappropriate medications, only 4.4% of NHs (*n* = 2) without such a pharmacist had such lists (*p*-value: 0.014). Similarly, 52.5% of NHs (*n* = 32) working with pharmacists in clinical roles had taken measures to reduce polypharmacy in residents, versus only 22.2% of NHs without (*n* = 10; *p*-value: 0.001). This pattern can be seen for all processes investigated (see Table 2 for details).

**Table 2:** Nursing home structures and processes based on pharmacist’s role

Structures and processes related to medication use and safety % yes (*n*)	NHs working with a pharmacist with a clinical role (*n* = 61)	NHs working with a pharmacist without a clinical role (*n* = 45)	Pearson Chi-square	*P *value*
List of preferred drugs for physicians	29.5 (18)	17.8 (8)	1.925	0.165
List of inappropriate medications	21.3 (13)	4.4 (2)	6.064	0.014
Electronic solutions for interactions check	72.1 (44)	68.9 (31)	0.132	0.717
Measures to reduce polypharmacy	52.5 (32)	22.2 (10)	10.370	0.001
Regular medication reviews	73.8 (45)	48.9 (22)	6.894	0.009

*Chi-Square Test of Independence. Valid *n* = 106. *P* value significance: 0.05

## Discussion

To our knowledge, this is the first study that systematically assessed medication use processes in nursing homes in Switzerland, inquiring specifically about organization, infrastructure and processes with a focus on interprofessional collaboration with pharmacists. As NHs primarily care for older people who have multiple co-morbidities and are often highly medicated because of it, medication safety is a critical issue in this context.

Many NHs have structures in place to guide and monitor medication safety with 91.5% having guidelines structuring medication use processes. Medication lists defining medications to be prescribed preferentially and PIM lists are well known, albeit their standardized use remains unclear.

The majority of Swiss NHs participating in this study have an electronic resident record system that could support medication safety, for instance by providing accessible, readable and unambiguous patient data and structured medication lists. 70.3% have electronic solutions available to check medication interactions. However, based on our results, we do not know whether and how these tools are used or strategically implemented (e.g., which staff member uses them, the frequency and format of use, whether it is automated or has alerts). These are key aspects of their impact [31–33]. Indeed, while electronic support of medication use processes is quite common in NHs, only a minority of NH leaders agree that their electronic resident record systems support adequate medication prescribing processes. This is also reflected in the literature, where the experience of end-users depends on functionality, content and structure of the electronic system [34]. This suggests that many of these systems could be either poorly-designed or poorly-used. Furthermore, clinical decision support features usually entail extra costs, which may impede their full implementation [35]. While the availability of structures (e.g., lists, electronic solutions) is encouraging, their value and process integration depend on how NH leadership and staff incorporate them into clinical practice [36].

Our results highlight that the implementation of electronic patient records is widespread, with electronic medication lists often available. However, interprofessional exchange across interfaces of care is still lacking. Even while the vast majority of Swiss NHs are currently collaborating with external health professionals, fewer than half (10.2–46.3%, depending on the partner) enable electronic data exchanges with those partners. In this study, the absence of a Swiss central health system is reflected in the differences between the French and German language regions’ integration of external partners in their electronic resident record systems.

While only 69.2% of included facilities were legally mandated to work with pharmacists, 87.1% were collaborating with pharmacies, suggesting that NHs increasingly deem such interprofessional collaboration beneficial. While pharmaceutical care models are increasingly implemented especially in French speaking regions of Switzerland[37], there is still a relative lack of clinical involvement of pharmacists in Swiss NHs overall, as reflected in our study. Those institutions working with pharmacists in clinical roles are more likely to conduct medication reviews and other actions to reduce polypharmacy than NHs that enlist them only for educational or logistical support. This indicates that access to clinical support by pharmacists improves safety-related processes. [24, 38]

However, if pharmacies lack access to resident data, their clinical tasks are more difficult to execute appropriately [39]. From a pharmacist’s perspective, increasing the opportunity to jointly access data, or at least increasing electronic documentation, would likely benefit interprofessional collaboration while optimizing medication-related processes and safety [40]. Indeed, the opportunity for electronic data exchange among the health care team and with external partners is an important prerequisite for interprofessional, coordinated care and timely, complete communication of medication-related information, which is one of the biggest risks in healthcare [41].

Beyond purely structural factors (e.g., case and skill-mix), interprofessional collaboration and access to physicians and pharmacists correlate with reduced MEs and are instrumental in the initiation and conduct of medication reviews [22]. Key implementation factors include care providers’ motivation and management commitment [42]. Our results show that several health professions are involved in medication reviews. For instance, pharmacists with a clinical role play a key part in medication reviews. Exploring ways to increase their clinical role in NHs (i.e., less than 60% had a clinical role in this study) might contribute to more deprescribing actions [43].

Overall, medication reviews appear to be executed regularly in a majority of NHs, which is a positive signal as they are important to the provision of high quality of care, and can also be an important de-prescribing step to reduce inappropriate polypharmacy and PIMs in NHs [44–46]. However, it is worth noting that we are not sure that NH leaders understand medication reviews as internationally defined [47]. Medication reviews were often reported to be performed during regular physician visits, which reflects international practice in the inpatient setting [48]. However, this is a complex undertaking in view of the time required. Indeed, at the time of our study, most medication reviews were undertaken during rounds, or reactively (following an event) but not proactively (anticipating medication-related risk, for example based on a validated assessment tool). While in our study, pharmacists were involved clinically only in a very limited capacity, the literature shows clearly that the integration of a clinical pharmacist in ward rounds and medication reviews is beneficial for residents’ medication safety [49]. Overall, time necessary to proactively, separately and interprofessionally carry out medication reviews is difficult to finance, making their integration in daily practice more challenging [50].

Furthermore, collaboration with nurses is likely to gain importance. Nurses can be quality partners for pharmacists, acting as liaison within the facility, for example to facilitate interprofessional collaboration or the initiation of proactive medication reviews [51]. However, it is not yet clear from the literature how pharmacists, physicians and nurses can best collaborate in undertaking medication reviews, or how tasks and responsibilities pertaining to medication use and safety should generally be allocated.

This study’s notable strengths include its large sample size and wide-ranging questions on medication safety processes and NH-pharmacy collaboration. Its weaknesses include the use of a convenience sample (sampling bias is possible), which limits the results’ generalizability and representativeness, and its cross-sectional design, which prevents us from concluding causal inferences. The use of self-reported data through surveys may have introduced certain response biases, such as social desirability bias, non-responder bias, and recall bias. Furthermore, despite the large sample size, group comparisons involve small group sizes (< 30). Consequently, these comparisons must be assessed with caution. Finally, the use of self-developed questions may limit the validity and reliability of our findings.

Further research should focus on how electronic resident record systems—and IT systems in general—can be used to better support medication use processes in NHs, e.g., to improve medication prescription, interprofessional collaboration, communication, and ultimately medication safety. Additionally, questions remain regarding the optimal set-up of medication review execution and which roles should be allocated to which health care professionals. Triggers for initiating medication reviews need to be developed and standardized. Finally, the current and potential roles and tasks of clinical pharmacists, and their potential positive impacts on quality of care and resident outcomes such as inappropriate polypharmacy, need to be further investigated.

## Conclusion

Most participating NHs had processes and structures in place to support adequate medication use and medication safety. While electronic patient records were common, the quality of integration has potential for improvement. Interprofessional collaboration was relatively common; although pharmacists played primarily logistical roles and their clinical involvement was limited. Optimizing electronic data access or at least exchange might be highly beneficial in furthering interprofessional collaboration. Nurses might be valuable partners for facilitating clinical interprofessional collaboration like initiating medication reviews. Further research is recommended on optimizing interprofessional collaboration in NHs, with a focus on resident medication reviews.
